# Relationship between hypercoagulability and mesenteric ischemia early after cardiac surgery

**DOI:** 10.1007/s11239-025-03186-z

**Published:** 2025-10-10

**Authors:** Zulfugar T. Taghiyev, Mike Sadowski, Lili-Marie Beier, Carina Leweling, Sophia Gunkel, Paula Keschenau, Johannes Kalder, Borros M. Arneth, Chrysanthi Skevaki, Ulrich Sachs, Jens Müller, Andreas Böning

**Affiliations:** 1 Department of Cardiovascular Surgery, Justus-Liebig-University Hospital, Giessen, Germany; 2 Institute of Laboratory Medicine and Pathobiochemistry, Molecular Diagnostics, Justus-Liebig-University Hospital, Giessen, Germany; 3 Institute of Laboratory Medicine and Pathobiochemistry, Molecular Diagnostics, Philipps University Hospital, Marburg, Germany; 4 Institute for Clinical Immunology and Transfusion Medicine, Justus-Liebig-University Hospital, Giessen, Germany; 5 Institute of Experimental Hematology and Transfusion Medicine, University Hospital Bonn, Medical Faculty, University of Bonn, Bonn, Germany

**Keywords:** Intestinal fatty acid-binding protein (I-FABP), Cardiac surgery, Hypercoagulability, Mesenteric ischemia, Thrombin-anti-thrombin complex (TAT), Prothrombin fragments 1+2 (F1.2)

## Abstract

Cardiac surgery is considered to be a hypercoagulable state with an increased incidence of thromboembolic events. To evaluate the connection between hypercoagulability and mesenteric ischemia (Me-Is), we investigated hemostatic parameters in patients with diagnosed Me-Is. Out of a cohort of 500 consecutive cardiac surgery patients, 25 patients with hyperinflammatory indicators (interleukin-6 > 600 ng/l) and metabolic acidosis (lactate > 4 mmol/l) were retrospectively matched 1:4 into Me-Is (*n* = 5) and control (*n* = 20) groups. Blood samples collected before surgery, on intensive care unit (ICU) admission, and 12 h after ICU admission were assessed for hemostatic parameters, including fibrinogen, D-dimer, thrombin-anti-thrombin complex (TAT), and prothrombin fragments 1 + 2 (F1.2). Thrombin generation assays were conducted on all samples, and intestinal fatty acid-binding protein (I-FABP) was assessed as a marker for Me-Is. Baseline levels of hemostatic markers were similar between the two groups. TAT levels were significantly higher in the Me-Is group 12 h after ICU admission (54.20 ± 10.49 vs. 22.18 ± 12.43 ng/ml, *p* = 0.010). In contrast, at ICU admission, absolute F1.2 values were higher in the control group (1.19 ± 0.04 vs. 0.49 ± 0.47 ng/ml, *p* = 0.047). However, increase of F1.2 values of the Me-Is group (394.2 ± 231.6%) vs. the control group (114.7 ± 144.9%) 12 h after ICU admission were 3.9- vs. 1.1-fold compared to baseline (*p* = 0.046). Postoperatively, higher levels of I-FABP and of D-dimers were observed in the Me-Is group at ICU admission (17116.2 ± 18185.4 vs. 2252.3 ± 1582.7 pg/ml; *p* = 0.006; and 5.3 ± 1.3 vs. 3.0 ± 2.1 µg/ml; *p* = 0.043; respectively) and 12 h after ICU admission (16998.2 ± 20346.3 vs. 1030.8 ± 1100.0 pg/ml; *p* = 0.030; and 3.7 ± 1.8 vs. 1.2 ± 0.8 µg/ml; *p* = 0.005; respectively) compared to the control group. No significant differences were observed for parameters of thrombin generation (TGA, peak value, ETP) between the two groups. Our findings suggest that TAT and F1.2 levels are promising candidates as markers of coagulability after cardiac surgery. High levels of activation markers suggest a temporary stage of hypercoagulability immediately after surgery in Me-Is patients. Nevertheless, the serial assessment of thrombotic profiles offers valuable mechanistic insights, although these exploratory findings require confirmation in larger cohorts.

**Supplementary Information:**

The online version contains supplementary material available at 10.1007/s11239-025-03186-z.

## Introduction

**Fig. 1:**
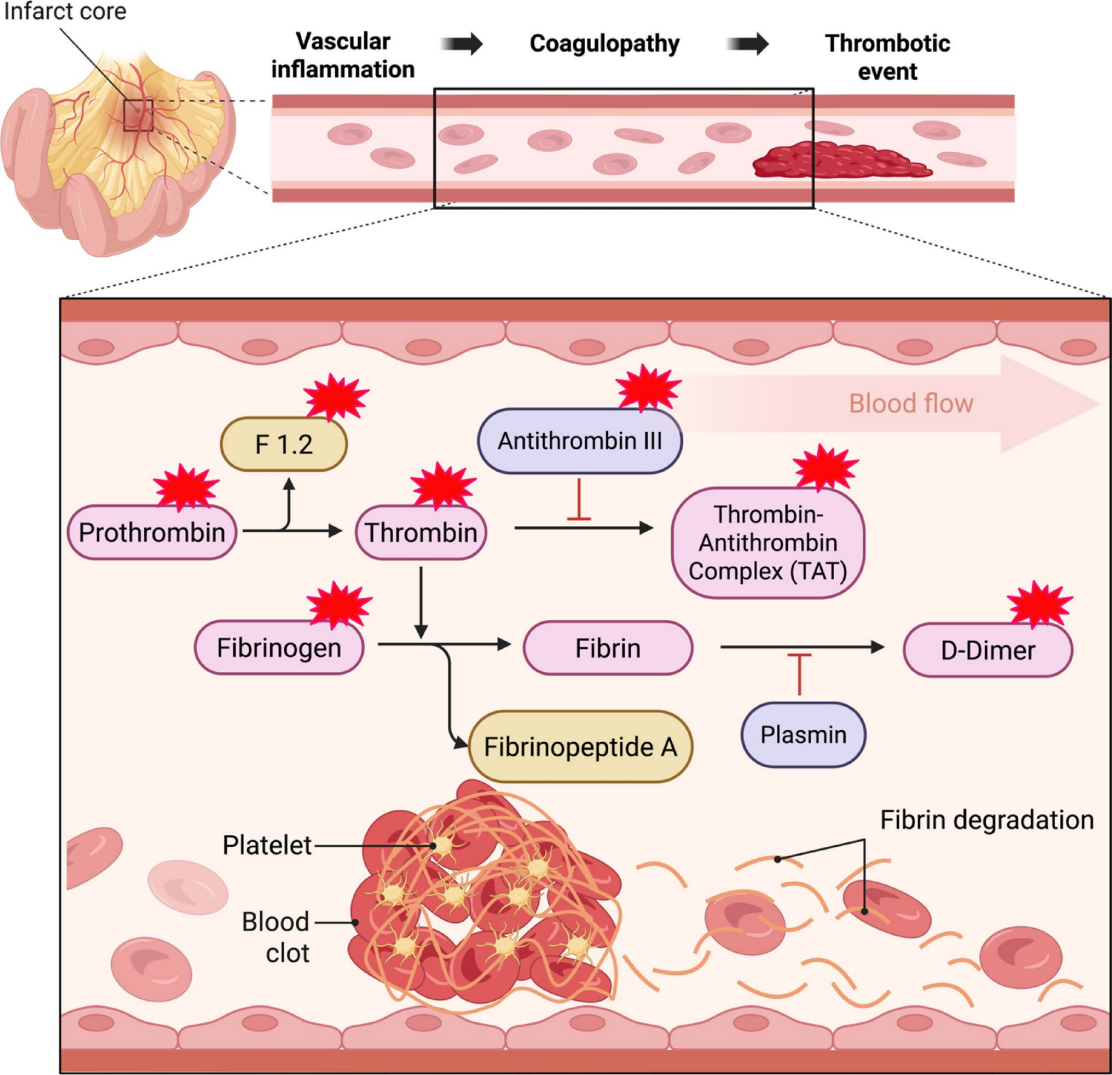
Coagulopathy at the site of mesenteric ischemia Pathophysiological relationship between vascular inflammation, coagulopathy, and thrombotic events. A vascular inflammatory reaction initiates coagulopathy with increased activation of the coagulation cascade. The conversion of prothrombin to thrombin leads to an increased formation of fibrin from fibrinogen, accompanied by a release of fibrinopeptide A. At the same time, there is an increased development of thrombin-antithrombin complex (TAT). The resulting fibrin thrombus is stabilized by platelet aggregation and can lead to vascular occlusion. The fibrinolytic system breaks down fibrin into D-dimers, which can thus serve as biomarkers for thrombotic events. The parameters marked with a red asterisk were analyzed in this study. (Created in BioRender. Taghiyev, T. (2025)

The connection between hypercoagulability and the occurrence of mesenteric ischemia (Me-Is) shortly after heart surgery can be traced back to a complex interplay of physical adaptation processes and consequences of stress due to surgery [1–4]. Although Me-Is is rare, it is one of the dreaded complications after cardiac surgery and is often associated with high mortality [5, 6]. Hypercoagulability is due to a pathologically increased formation of thrombin that can trigger uncontrolled fibrin deposits or increased platelet aggregation (Fig. 1). Due to the formation of microthromboemboli in the intestinal vessels, this overactivity of the coagulation system can restrict blood flow to the intestine to such an extent that tissue damage occurs [7, 8].

In order to avoid consequential damage from Me-Is, rapid detection and targeted treatment are crucial. As studies by Eris and colleagues (2013) show, both the physical stress response during surgery and the use of the heart-lung machine can further increase the clotting tendency [2]. However, current data suggest that early anticoagulant therapy could improve patients’ prognosis [9].

Thus far, it is unclear whether an increased potential for thrombin formation increases the risk of vascular complications and Me-Is after cardiac surgery. In order to measure this potential, so-called thrombin formation tests (carried out in vitro or ex vivo) are used, the reliability of which has already been confirmed in several studies [10, 11]. Conventional coagulation tests often do not adequately capture the actual risk of thrombosis in Me-Is, which is why these special assays play a key role. In order to estimate thrombin activity directly in the body, the prothrombin fragments 1 + 2 (F1.2), a degradation product produced during the conversion of prothrombin to thrombin, is often analyzed [12, 13]. Thrombin-antithrombin complexes (TAT), which form when thrombin is neutralized by antithrombin (Fig. 1), are also significant.

The present study investigated the extent to which hypercoagulability after cardiac surgery is related to Me-Is and how this coagulation disorder affects thrombin formation.

## Patients and methods

### Ethics statement

The study was approved by the Ethics Committee of Justus Liebig University Giessen (Local Registration Number: GI AZ 293/20, Amendment 2 (12.08.2022) and has a unique identifier on www.clinicaltrials.gov: NCT06365827. All participants gave their written consent to participate in this prospective registry study.

### Screening and patient cohort

This prospective observational study included 500 out of 929 consecutive patients who underwent open-heart surgery at a clinic between March 2022 and December 2023. Patients with chronic organ insufficiency, preoperative infections (e.g., endocarditis), severe immunodeficiency, or lack of consent were excluded. Anonymized medical data were recorded electronically and evaluated using REDCap (Research Electronic Data Capture). The present study adheres to the STROBE (Strengthening the Reporting of Observational Studies in Epidemiology) guidelines for observational research, with specific consideration given to the combined design structure—namely, a nested case-control study embedded within a prospective cohort. Detailed methodological transparency is provided in accordance with the STROBE checklist (**Supplementary STROBE Statement**) [14].

### Target population

**Fig. 2:**
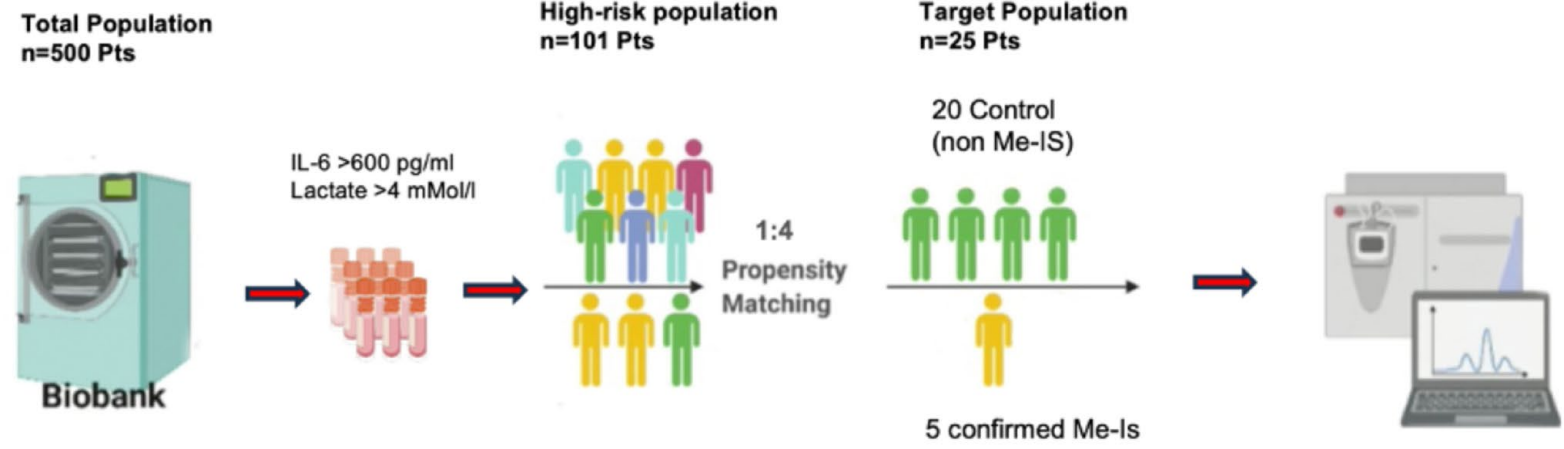
Overview of the total and target populations From a biobank of 500 surgical patients, 101 high-risk individuals (IL-6 > 600 pg/mL, lactate > 4 mmol/L) were identified. Five patients with confirmed mesenteric ischemia (Me-IS) were matched to 20 non-Me-IS controls (1:4 ratio). (Created in BioRender. Taghiyev, T. (2025) Abbreviations: Me-Is = mesenteric ischemia; IL-6 = Interleukin-6

Of 500 consecutive patients, 6 (1.2%) showed confirmed Me-Is. One patient was excluded from the analysis due to non-occlusive Me-Is (NOMI) diagnosed early after admission to the intensive care unit (ICU) and was treated with prostaglandin perfusion. Of 101 high-risk patients with hyperinflammatory status (interleukin-6 [IL-6] >600 ng/l) and metabolic acidosis (lactate >4 mmol/l), a target population of 25 were assigned by propensity matching to a Me-Is group (*n* = 5) or a control group (*n* = 20) in a ratio of 1:4. The combined use of lactate ≥ 4 mmol/L and IL-6 concentrations of approximately 600 pg/mL as inclusion thresholds is supported by previous studies, demonstrating their prognostic value in critically ill patients [15–18]. This ensured that the study cohort represented patients with severe metabolic and inflammatory dysregulation at high risk of adverse outcomes. In the Me-Is group, all five patients underwent laparotomy 2–3 days after ICU admission because of clinical signs of peritonitis, and the diagnosis of mesenteric ischemia was confirmed intraoperatively and subsequently by histopathological examination. Baseline characteristics of the overall and target populations are presented in Table 1, while Table 2 provides the intraoperative characteristics and the matched comparison of the Me-Is and control groups. The study population was thus divided into two groups: Me-Is and non-Me-Is controls (Fig. 2).

### Collection of samples and laboratory measurements

**Fig. 3:**
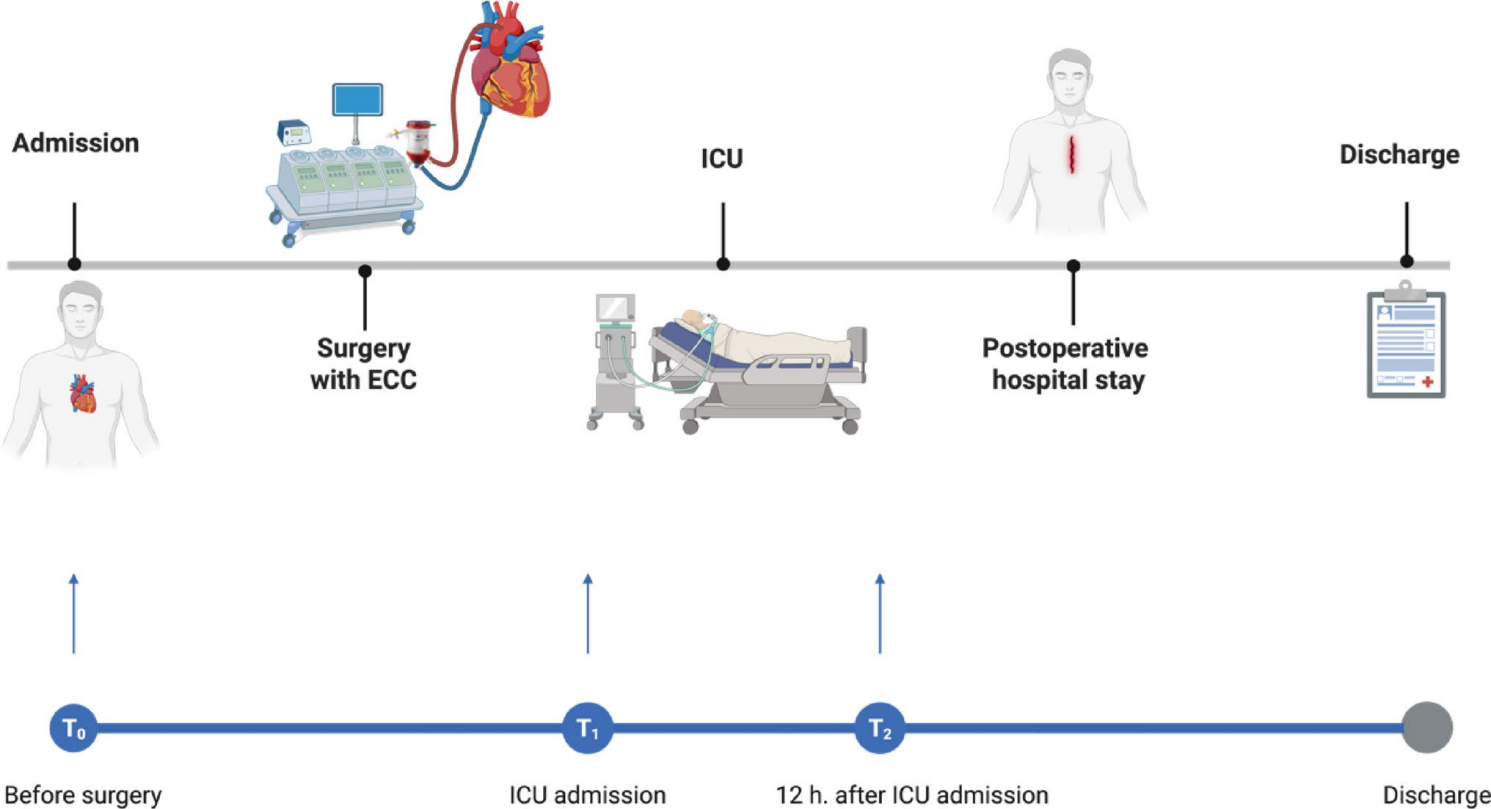
Overview of sampling time points of citrat-blood samples Patients undergoing cardiac surgery with extracorporeal circulation (ECC) were monitored from admission through discharge. Blood samples were collected at T₀ (pre-surgery), T₁ (ICU admission), and T₂ (12 h post-ICU admission) to enable perioperative analysis. (Created in BioRender. Taghiyev, T. (2025). Abbreviations: ECC = Extracorporeal Circulation; ICU = Intensive Care Unit; T_0_: Preoperative timepoint (before surgery); T_1_: Timepoint at ICU admission; T_2_ 12 h after ICU admission

Sampling and laboratory measurements of the citrate blood samples were taken perioperatively at 3 time points: T0 – preoperatively; T1 – upon admission to the ICU; T2 – 12 hours after ICU admission. An overview is given in Figure 3. 

Blood was collected in citrate tubes and centrifuged twice (2000 × g, 10 min, at room temperature) to obtain platelet-poor plasma. Plasma was carefully removed, aliquoted, and immediately stored at −80 °C. Before analysis, rapid thawing (37 °C water bath), careful mixing, and storage at 4 °C until examination (maximum 2 h after thawing) were carried out.

For quality control, visual assessments of the platelet-free supernatant were made after each centrifugation step. The samples were immediately frozen to protect labile biomarkers. Polypropylene tubes were used to minimize adsorption.

For the quantitative determination of the prothrombin fragment F1.2 in human plasma, the Enzygnost™ F1 + 2 (monoclonal) assay from Siemens Healthineers (Marburg, Germany) was used. This enzyme-based immunological test is used to diagnose, monitor, and evaluate acquired or hereditary blood clotting disorders and supports the risk assessment for thrombosis and the monitoring of the effectiveness of anticoagulants.

The concentration of TAT was determined using the Enzygnost™ TAT microassay from Siemens Healthineers. This ELISA test enables the quantitative determination of TAT complexes in plasma and is used to diagnose hypercoagulability conditions such as disseminated intravascular coagulopathy (DIC).

Thrombin formation was analyzed with the RC Low reagents on the Ceveron^®^ s100 system of Technoclone (Vienna, Austria). The Ceveron^®^ s100 is a fully automated system designed to perform coagulation, chromogenic, and turbidimetric assays as well as thrombin generation testing (TGA) and quenching assays. The RC Low reagents have been specially developed for the investigation of thrombophilic tendencies and enable a detailed analysis of thrombin formation.

### Statistical analysis

The statistical analyses were conducted utilizing Statistical Package for the Social Sciences (SPSS^®^) version 27.0 for Mac OS (IBM^®^ Corporation released 2019, Armonk, New York, United States) and GraphPad Prism version 9.0.0 for Mac OS (GraphPad Software released 2020, San Diego, California USA) following appropriate coding procedures. Continuous variables are expressed as mean ± standard deviation (SD), and categorical variables are presented as frequencies and percentages. Inter-group disparities across various time points were assessed employing one-way analysis of variance (ANOVA), with Tukey’s post hoc test being applied in instances of observed differences. The normality of data distribution within each group was evaluated using the Shapiro-Wilk test. For normally distributed variables, Student’s t-test (unpaired) was utilized for comparison, and non-normally distributed variables were subjected to analysis using either the Mann-Whitney U-test or the Wilcoxon-signed-rank test.

Comparisons between different groups were made employing Pearson’s chi-squared test or Fisher’s exact test to ascertain independence of measurements. A standard confidence level of 95% was set, and statistical significance was determined at a p-value less than 0.05 (two-tailed). In instances of multiple comparisons, adjustments were made utilizing the Bonferroni correction method. Furthermore, the handling of outliers has been clarified, indicating that no extreme values were excluded from the analyses.

For propensity score matching, perioperative risk variables including age, body mass index (BMI), sex, EuroSCORE II, platelet count, partial thromboplastin time (PTT), and IL-6 and lactate levels were used to compare the two groups and minimize selection bias. Matching was performed at a 1:4 ratio (5 Me-Is patients vs. 20 control patients) based on propensity scores, without replacement, in order to minimize baseline differences and reduce the risk of selection bias.

No formal correction for multiple testing was performed, as the study was designed with an exploratory character and primarily aimed to generate hypotheses.

## Results

### Demographic data

The initial characteristics of the total population (*n* = 500) and the target population (*n* = 25) are shown in Table 1. In the target group, the proportion of female patients was nominally higher (36%) than in the total population (26%), although this was not statistically significant (*p* = 0.291). The target population was significantly older than the total population (69.80 ± 5.95 vs. 53.50 ± 26.72 years; *p* = 0.003), and there was a higher prevalence of heart failure NYHA class III–IV (64% vs. 30%; *p* < 0.001). Patients in the target population also showed a significantly lower glomerular filtration rate (eGFR) (74.5 ± 27.1 vs. 84.8 ± 26.0 ml/min/1.73 m²; *p* = 0.001) and a significantly higher EuroSCORE II (10.92 ± 9.9 vs. 4.06 ± 3.6; *p* = 0.001). Other variables did not differ significantly between the two groups.

**Table 1:** Baseline characteristics of total and target populations

Variable, *n* (%) or mean ± SD	Total Population	Target Population	*p* Value	
	(*n* = 500)	(*n* = 25)		
**Female**,** n (%)**	132 (26)	9 (36)	0.291	
**BMI**,** kg/m** ^2^	28.11 ± 5.05	29.25 ± 5.45	0.273	
**Age**,** y**	53.50 ± 26.72	69.80 ± 5.95	0.003	
**COPD Gold III-IV**,** n (%)**	22 (4.4)	2 (8)	0.400	
**NYHA class III- IV**,** n (%)**	152 (30)	16 (64)	0.000	
**CCS class III-IV**,** n (%)**	66 (13.2)	4 (16)	0.688	
**Diabetes on insulin**,** n (%)**	46 (9.2)	5 (20)	0.075	
**HbA1c**,** %**	5.98 ± 0.95	6.07 ± 0.78	0.642	
**Ejection fraction**,** %**	53.3 ± 11.14	50.2 ± 10.65	0.174	
**Serum creatinine**,** mg/dl**	1.0 ± 0.6	1.2 ± 1.2	0.128	
**eGFR**,** ml/min/1.73 m**^2*^	84.8 ± 26.0	74.5 ± 27.1	0.001	
**STS-Prom score**,** predicted mortality**,** %**	5.29 ± 7.22	6.85 ± 7.95	0.295	
**EuroSCORE II**	4.06 ± 3.6	10.92 ± 9.9	0.001	

*Cockcroft-Gault Equation [19]

Abbreviations: BMI = Body Mass Index; COPD GOLD = Chronic Obstructive Pulmonary Disease, Global Initiative for Chronic Obstructive Lung Disease Stages; NYHA = New York Heart Association Functional Classification; CCS = Canadian Cardiovascular Society Angina Grading Scale; HbA1c = Glycated Hemoglobin; eGFR = Estimated Glomerular Filtration Rate; STS-PROM Score = Society of Thoracic Surgeons Predicted Risk of Mortality; EuroSCORE II = European System for Cardiac Operative Risk Evaluation II

After adjusting the baseline variables by propensity score matching, there were no significant differences between the Me-Is group (*n* = 5) and the control group (non-Me-Is; *n* = 20) (Table 2). Preoperative features were comparable between the two groups. Furthermore, no patient in either group had a documented pre-existing coagulopathy. Intraoperatively, the two groups also did not differ significantly in terms of combined procedures, duration of extracorporeal circulation (CPB time), and duration of aortic clamping. Postoperatively, there tended to be a higher frequency of the need for mechanical support systems in the Me-Is group compared to the control group (60% vs. 25%; *p* = 0.134), but this difference was not significant (Table 2).

**Table 2:** Baseline characteristics of the two groups from the propensity-matched population

Variable, *n* (%) or mean ± SD	Me-Is	Non Me-Is	*p* Value
	(*n* = 5)	(*n* = 20)	
Preoperative characteristics			
Male, n (%)	4 (80)	11 (55)	0.307
BMI, kg/m^2^	28.2 ± 6.2	30.3 ± 4.7	0.455
Age, y	68.7 ± 4.3	70.8 ± 7,6	0.324
Ejection fraction, %	51.7 ± 10.4	48.6 ± 10.9	0.589
STS-Prom score, predicted mortality, %	6.5 ± 6.8	7.2 ± 9.1	0.874
EuroSCORE II	11.5 ± 10.4	10.5 ± 9.3	0.835
eGFR, mL/min/1.73 m^2*^	76.1 ± 27.2	72.9 ± 26.9	0.814
HbA1c, %	5.7 ± 1.4	6.1 ± 0.7	0.360
**Intraoperative characteristics**			
Combined surgery, n (%)	2 (40)	11 (55)	0.548
CPB time, mean ± SD, hh: mm	2:39 ± 0:43	3:18 ± 0:50	0.626
Cross-clamp time, mean ± SD, hh: mm	1:30 ± 0:30	1:29 ± 0:35	0.871
**Postoperative characteristics**			
Need for assist devices (n, %)	3 (60)	5 (25)	0.134
ICU stay, days	5.6 ± 6.2	8.7 ± 8.5	0.455
Invasive ventilation, h	39.2 ± 28.1	61.2 ± 89.6	0.598
Dialysis, n (%)	1 (20)	4 (20)	1.000
Re-thoracotomy, n (%)	2 (40)	2 (10)	0.102
APACHE II score	23.7 ± 7.6	22.9 ± 3.3	0.717
SOFA score	9.0 ± 2.7	8.4 ± 2.6	0.651
Norepinephrine support, µg/kg	412.8 ± 494.9	507.3 ± 813.4	0.807

*Cockcroft-Gault Equation [19]

Abbreviations: BMI = Body Mass Index; STS-PROM Score = Society of Thoracic Surgeons Predicted Risk of Mortality; EuroSCORE II = European System for Cardiac Operative Risk Evaluation II; eGFR = Estimated Glomerular Filtration Rate; HbA1c = Glycated Hemoglobin; CPB *=* Cardiopulmonary Bypass; ICU = Intensive Care Unit; APACHE II *=* Acute Physiology and Chronic Health Evaluation II; SOFA **=** Sequential Organ Failure Assessment

The initial laboratory values of both groups are shown in Table 3. Patients with Me-Is tended to have higher D-dimer levels (1.5 ± 0.8 mg/L vs. 0.8 ± 0.9 mg/L; *p* = 0.130) and lower platelet counts (248.0 ± 60.7 per µL vs. 459.8 ± 912.9 per µL; *p* = 0.615) compared to the control group, although these differences were not statistically significant.

**Table 3:** Baseline labor characteristics of target population

Variable, *n* (%) or mean ± SD	Me-Is (*n* = 5)	Non-Me-Is (*n* = 20)	*p* Value
Prothrombin time (Quick), sec	93.0 ± 19.9	97.3 ± 17.4	0.639
Partial thromboplastin time (PTT), sec	29.8 ± 3.3	30.45 ± 3.7	0.726
International normalized ratio (INR)	1.1 ± 0.1	1.0 ± 0.2	0.826
Fibrinogen, g/L	4.3 ± 1.5	3.8 ± 1.0	0.375
D-dimer, mg/L	1.5 ± 0.8	0.8 ± 0.9	0.130
Calcium, mmol/L	2.2 ± 0.2	2.3 ± 0.1	0.162
Platelet count, pro µL	248.0 ± 60.7	459.8 ± 912.9	0.615
Antithrombin III (ATIII), %	102.5 ± 10.6	105.4 ± 9.7	0.604

**Fig. 4:**
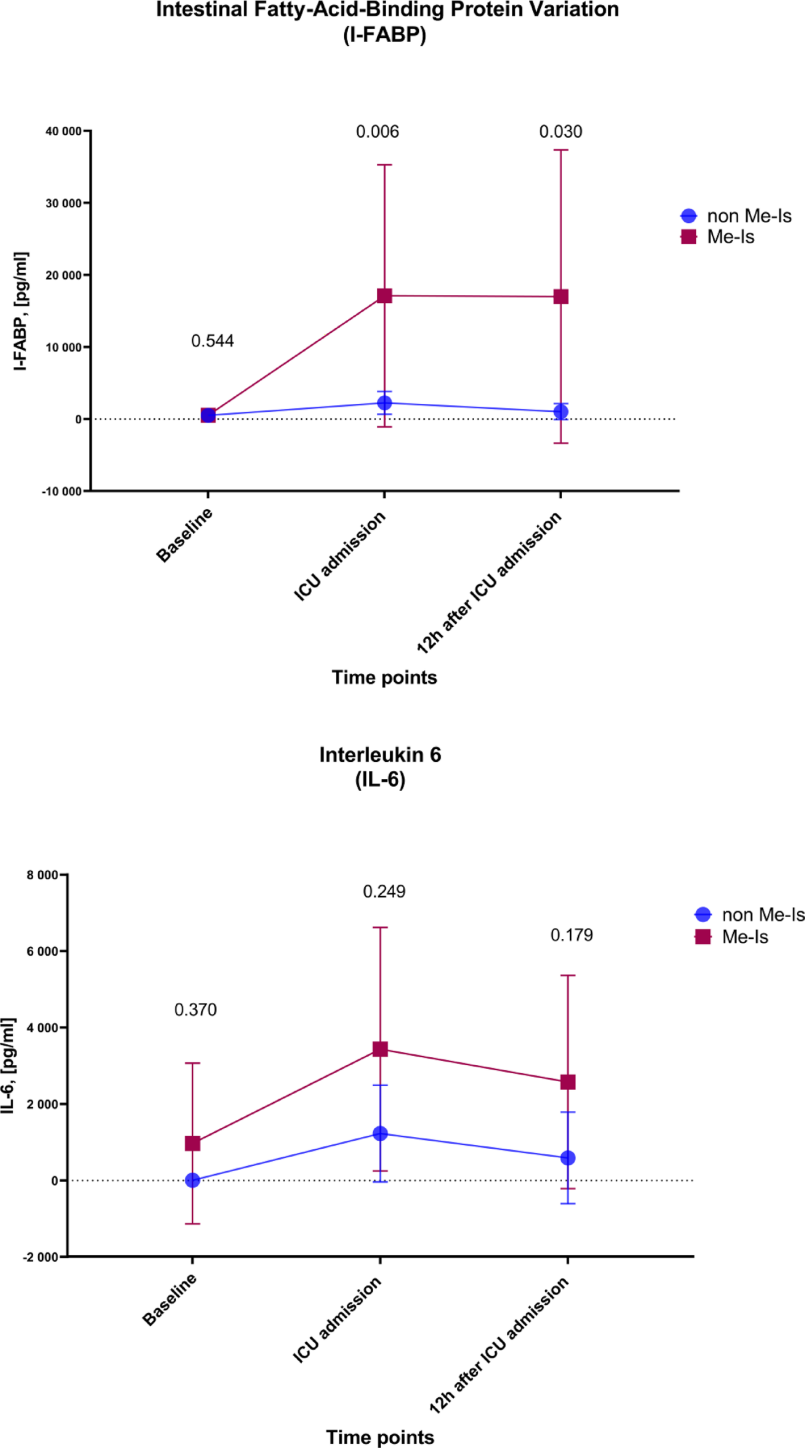
Time course of serum concentrations of the intestinal fatty acid-binding protein (I-FABP, above) and interleukin-6 (IL-6, below) in the group with mesenteric ischemia (Me-Is) compared to the control group (non-Me-Is). Data are presented as mean ± SD. Statistical significance is indicated by p-values (*p* < 0.05)

Figure 4 shows the time course of the concentrations of intestinal fatty acid-binding protein (I-FABP) and IL-6 between the group with Me-Is and the control group (non-Me-Is). In patients with Me-Is, there was a significant increase in I-FABP concentration at the time of admission to the ICU compared to the control group (2252.25 ± 1582.69 pg/ml vs. 17116.20 ± 18185.41 pg/ml, 95%CI [−22847.99 to −6879.90], *p* = 0.006). This difference remained significant even 12 h after ICU admission (1030.79 ± 1099.92 pg/ml vs. 16998.15 ± 20346.39 pg/ml, 95%CI [−24804.38 to −7130.34], *p* = 0.030).

For interleukin-6 (IL-6), there was initially no significant difference between the two groups at ICU uptake (1100.14 ± 1207.94 pg/ml vs. 2933.50 ± 3019.41 pg/ml, 95%CI [−5544.49 to 1877.77]; *p* = 0.330). However, 12 h after ICU admission, the IL-6 concentration was higher in the Me-Is group than in the control group (533.57 ± 1107.23 pg/ml vs. 2585.86 ± 2801.84 pg/ml, 95%CI [−5498.10 to 1393.52]), but this difference did not reach statistical significance (*p* = 0.179) (Fig. 4).

**Fig. 5:**
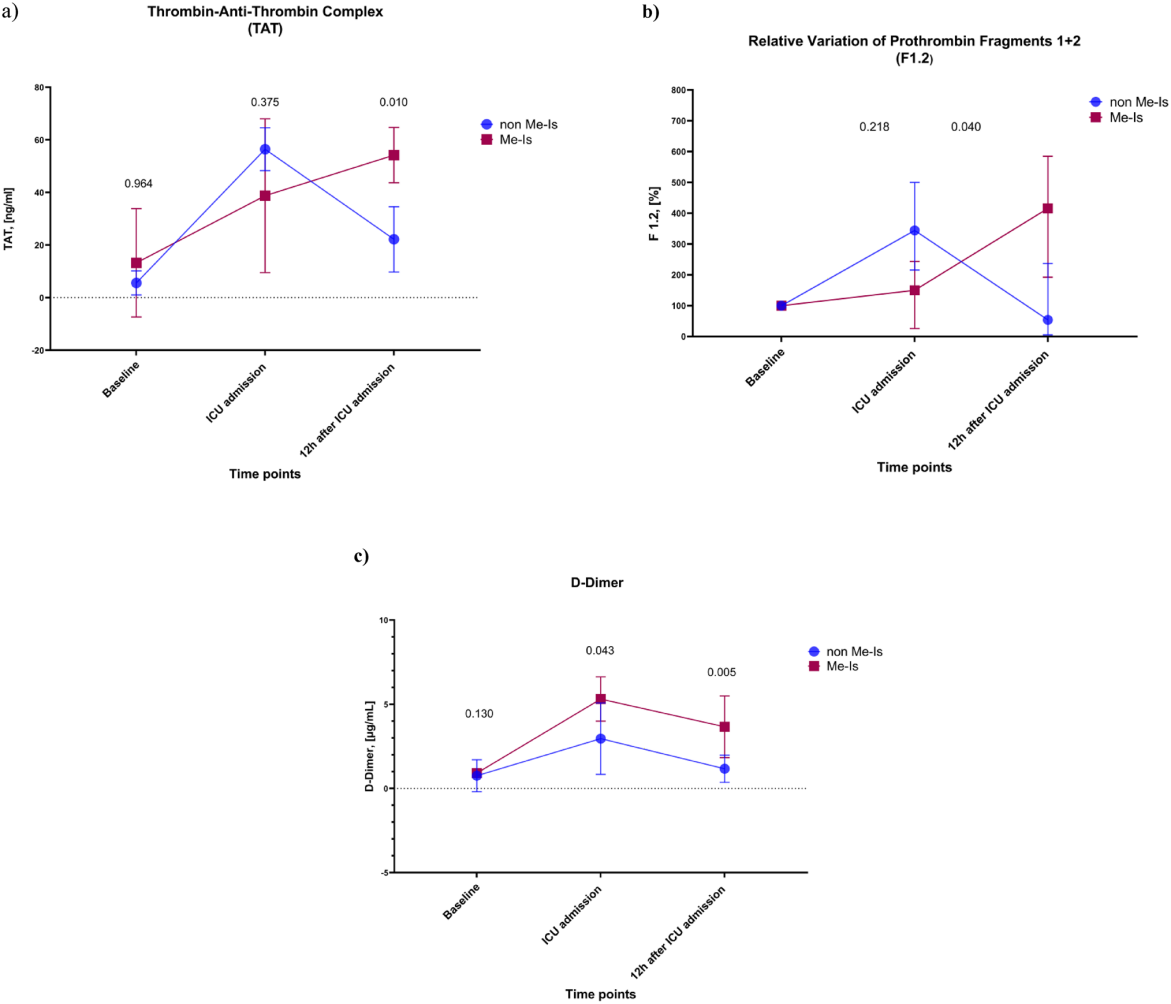
Time course of the levels of the enzymatic coagulation activation markers thrombin-antithrombin complex (TAT, a), prothrombin fragments 1 + 2 (F1.2, b) and D-dimers (c). Data are presented as mean ± SD. Statistical significance is indicated by p-values (*p* < 0.05)

Figure 5 shows the time course of the enzymatic coagulation activation markers TAT, prothrombin fragments 1 + 2 (F1.2), and D-dimers. TAT did not show any significant differences between the groups at the time of ICU admission (*p* = 0.375). However, 12 h after ICU admission, the TAT value was significantly higher in the Me-Is group than in the control group (54.20 ± 10.49 vs. 22.18 ± 12.43 ng/ml, 95%CI [7.46 to 38.50], *p* = 0.010)

In contrast, the absolute values of F1.2 on admission to the ICU were significantly higher in the control group than in the Me-Is group (1.19 ± 0.04 vs. 0.49 ± 0.47 ng/ml, 95%CI [−0.57 to 1.44], *p* = 0.047). However, the relative changes in F1.2 concentrations within 12 h of ICU admission showed a 3.9-fold increase from baseline in the Me-Is group (394.2 ± 231.6%), compared to only a 1.1-fold increase in the control group (114.7 ± 144.9%). This difference was statistically significant (95%CI [−448.45 to −110.54], *p* = 0.046).

The concentrations of D-dimers were already significantly higher in the Me-Is group at admission to the ICU (5.3 ± 1.3 vs. 3.0 ± 2.1 µg/ml, 95%CI [−4.46 to −0.25], *p* = 0.043). This difference was even more pronounced 12 h after ICU admission (3.7 ± 1.8 vs. 1.2 ± 0.8 µg/ml, 95%CI [−3.66 to −1.34], *p* = 0.005). In addition, there was also a significantly increased D-dimer level in the Me-Is group compared to the control group (**Supplementary Fig. 1b**). Taken together, these results suggest that patients with Me-Is have more pronounced activation of clotting.

**Fig. 6:**
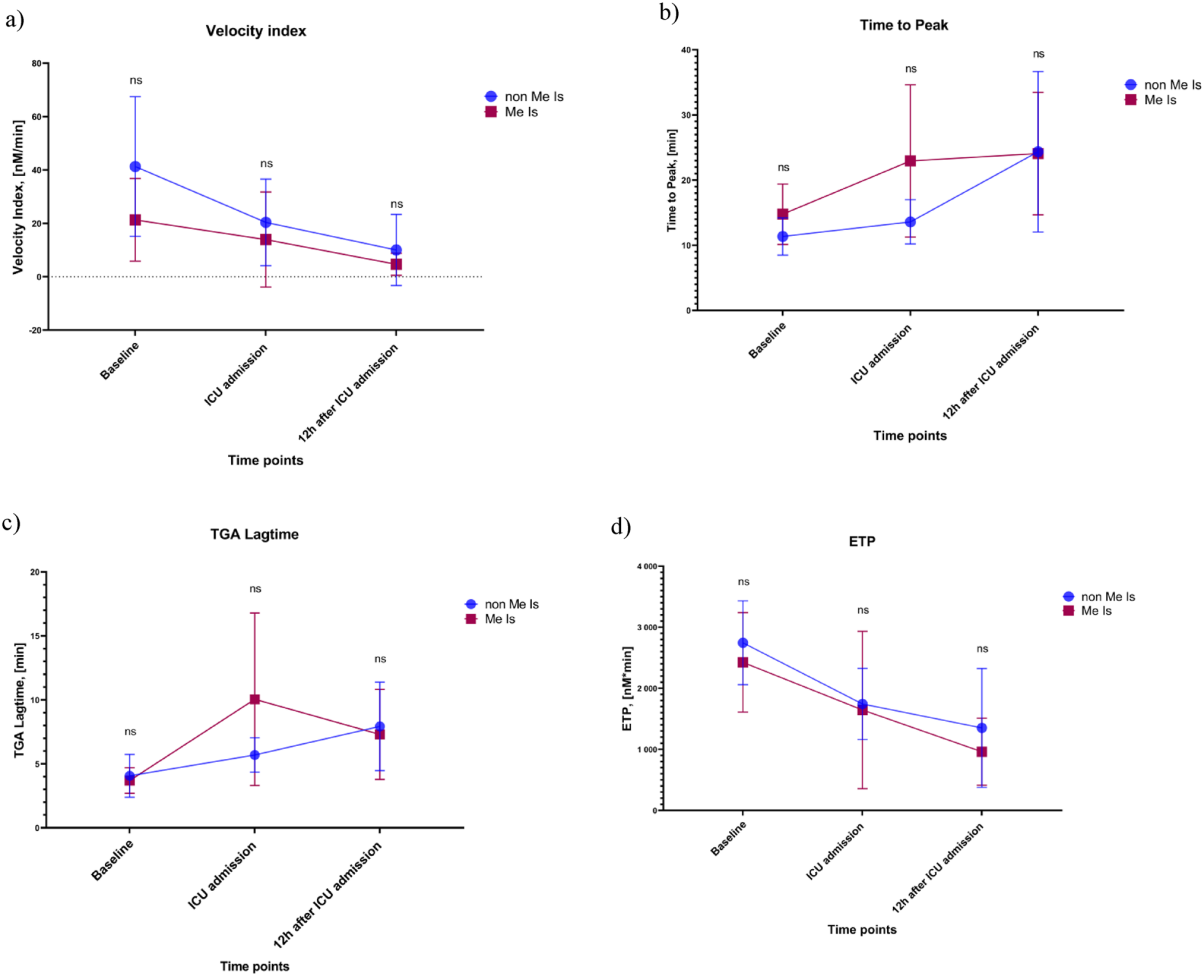
The results of the thrombin generation test (TGA) over time. (**a**) Velocity index; (**b**) Time to peak; (**c**) TGA lag-time; (**d**) ETP- Endogenous Thrombin Potential. Data are presented as mean ± SD. Statistical significance is indicated by p-values (*p* < 0.05)

Parameters characterizing thrombin generation (velocity index, time to peak, lag-time, and endogenous thrombin potential (ETP)) showed no significant differences over time between patients with Me-Is and the control group (non-Me-Is) (Fig. 6). Thus, there was no evidence of an association between the occurrence of Me-Is and altered thrombin-generating parameters.

The time course of the levels of the coagulation markers fibrinogen, partial thromboplastin time (PTT), antithrombin III (ATIII), and platelet count are shown in **Supplementary Figs. 1 and 2**. While there were no significant differences between the two groups for fibrinogen, which is an indicator of coagulation activation and acute phase reaction, PTT, a parameter of the intrinsic coagulation cascade, was significantly prolonged in the Me-Is group from 36 h after admission to the ICU. Antithrombin III (ATIII), which reflects physiological anticoagulation and hepatic synthesis performance, was significantly reduced in the Me-Is group as early as 12 h after ICU admission. Overall, these results suggest increased coagulation activation, consumption coagulopathy, and relevant ATIII consumption later in patients with Me-Is. The platelet count remained unchanged throughout the course in both groups.

## Discussion

Acute Me-Is is a life-threatening condition characterized by a sudden closure of the mesenteric blood supply that leads to intestinal ischemia and potential tissue necrosis. In this setting, ischemia-induced endothelial dysfunction disrupts these processes, resulting in a pro-inflammatory and pro-thrombotic state [20]. In addition, ischemia causes impairment of endothelial barrier function, allowing for increased extravasation of plasma proteins and inflammatory cells into the intestinal tissue. This barrier disorder promotes the formation of microthrombi within the microcirculation, which in turn further impairs blood flow and thus exacerbates ischemia [20, 21].

Damage to the vascular endothelium triggers prothrombotic mechanisms: thrombin is formed in excess, favored by exposed tissue factor and a loss of inhibitory counterregulation, which leads to fibrin-rich microthrombi in the microcirculation [22]. This dysfunction of the microcirculation aggravates local ischemia and can initiate systemic disseminated intravascular coagulation (DIC) in severe cases. Fibrin microthrombi are often found in the capillaries of ischemic-necrotic intestinal tissue upon histopathological analysis [23], but these are considered a non-specific expression of tissue necrosis and are not initially to be equated with manifest DIC [23].

Our analyses showed that in patients with Me-Is, early laboratory assessment can typically detect hypercoagulability, which is characterized by increased coagulation marker values. In particular, there was a significant increase in F1.2, which is considered a sensitive biomarker for increased thrombin formation in vivo. At the same time, increased TAT was observed, which serves as a substitute for active thrombin formation and binding. The elevation in levels of D-dimer, a specific degradation product of fibrin, is an indication of fibrin-mediated coagulation activation with consecutive fibrinolysis. This constellation of increased thrombin formation markers and fibrinolysis products reflects the massive thrombin generation and clot formation in Me-Is. It is noteworthy that despite the increased coagulation activation, the measured levels of thrombin and the results of the thrombin generation assay (TGA) remained unchanged. The fibrinogen level also remained initially within the normal range, which indicates an absence of consumptive coagulopathy – so there are no signs of significant coagulation factor consumption yet. Clinically, this pattern of findings corresponds to an early hypercoagulative phase without manifest DIC; i.e., a subclinical hypercoagulopathy in which increased thrombin formation takes place but no systemic consumption coagulopathy has (yet) occurred. This interpretation should be regarded as a plausible mechanistic explanation rather than definitive evidence and therefore remains speculative, warranting confirmation in larger patient cohorts.

Initially, upon ICU admission, the measured concentrations of interleukin-6 revealed no statistically meaningful distinction between the Me-Is group and the controls (*p* = 0.330). Nevertheless, at 12 h post-admission, IL-6 levels were markedly elevated in patients diagnosed with Me-Is compared to the control cohort, although this difference was not statistically significant (*p* = 0.179). This temporal progression underscores a delayed yet notably heightened systemic inflammatory reaction, characterized by an increase in pro-inflammatory cytokines such as IL-6, suggesting an exacerbation of underlying intestinal pathology over time. The data thus indicate more pronounced intestinal damage and inflammatory response in patients with Me-Is.

In terms of therapy, early anticoagulation (e.g., with unfractionated heparin) appears to be useful at this stage in order to counteract microvascular thrombosis and prevent progression to DIC [24, 25]. It is obvious that the thrombosis prophylaxis strategy currently used, in particular the administration of low-molecular-weight heparin, is not optimal. Therefore, there is a need for improved thrombosis prophylaxis. A better understanding of the pathophysiological mechanisms of thrombus formation in different situations could provide new preventive targets and lay the foundation for more effective prophylaxis strategies [26]. On the other hand, intensified anticoagulation is always a double-edged sword in postoperative patients, as it potentially increases the risk of bleeding and thus requires careful benefit-risk assessment.

Although hypercoagulability plays an essential role in the development of Me-Is after cardiac surgery [27], it is not the only factor. Other influencing variables such as the surgical stress response and pre-existing diseases are also critical [2]. In this context, Oldenburg et al. (2004) emphasized the clinical relevance of cardiac conditions and surgical stress as important contributors to acute mesenteric ischemia, while Becker (2020) described the pathophysiological mechanisms of coagulopathy and microvascular thrombosis, which may also affect mesenteric circulation. Together, these works support the biological plausibility of postoperative coagulation activation as a contributing factor in mesenteric ischemia [4]– [28]. The complexity of this interplay underlines the need for a multidisciplinary approach to diagnostics and therapy that should be aimed in particular at early detection and intervention in order to reduce the high mortality rate in this clinical scenario.

Our findings suggest that TAT and F1.2 levels are promising candidate markers for evaluating coagulability after cardiac surgery. High levels of activation markers suggest a temporary stage of hypercoagulability immediately after surgery in Me-Is patients. Given the limited sample size, these results should be regarded as preliminary and hypothesis-generating, and the potential role of TAT and F1.2 as early markers of mesenteric ischemia requires confirmation in larger studies. At the same time, the serial assessment of thrombotic profiles nonetheless provides valuable mechanistic insights and may help to inform future therapeutic strategies. Accordingly, the present findings cannot be translated into clinical practice at this stage and should be interpreted solely as exploratory.

## Study limitations

This study has several limitations. It relies on a non-randomized analysis of prospectively collected data from a single center, and the small size of the verified mesenteric ischemia subgroup (*n* = 5) substantially limits statistical robustness. In addition, there is a potential for selection bias in the control population, which needs to be considered when interpreting the results. The primary limitation of the study is the small sample size. However, the serial assessment of thrombotic profiles provides valuable insights into the underlying mechanisms of mesenteric ischemia and may help to guide the development of optimal therapeutic strategies. Nevertheless, the serial assessment of thrombotic profiles provides valuable mechanistic insights and may help to inform future therapeutic strategies. Furthermore, the exploratory and hypothesis-generating nature of this work must be emphasized, as confirmation of the observed associations requires validation in larger, independent cohorts. Accordingly, the clinical applicability of the proposed markers is limited at this stage.

## Supplementary Information

Below is the link to the electronic supplementary material.

Supplementary Material 1

Supplementary Material 2

## Data Availability

The article’s data will be shared on reasonable request to the corresponding author.

## Abbreviations

ANOVA: Analysis of variance
BMI: Body mass index
BL: Baseline
CPB: Cardiopulmonary bypass
CrCl: Creatinine clearance
eGFR: Estimated glomerular filtration rate
ICU: Intensive care unit
LVEF: Left ventricular ejection fraction
Me-Is: Mesenteric ischemia
SD: Standard deviation
STS Prom: Society of Thoracic Surgeons Predicted Risk of Mortality

## Declarations

### Clinical trial registration number

Ethics Committee of the Justus Liebig University Giessen - Local Registration Number: GI AZ 293/20, Amendment 2 (12.08.2022); www.clinicaltrials.gov unique identifier: NCT06365827.
