# Supplementary material for: Relationship between hypercoagulability and mesenteric ischemia early after cardiac surgery
Source: J Thromb Thrombolysis. 2025 Oct 10;59(2):494–505. doi: 10.1007/s11239-025-03186-z (PMC13018050; doi:10.1007/s11239-025-03186-z)
Supplement: Supplementary file 2 — Supplementary Material 2 [file 11239_2025_3186_MOESM2_ESM.docx]

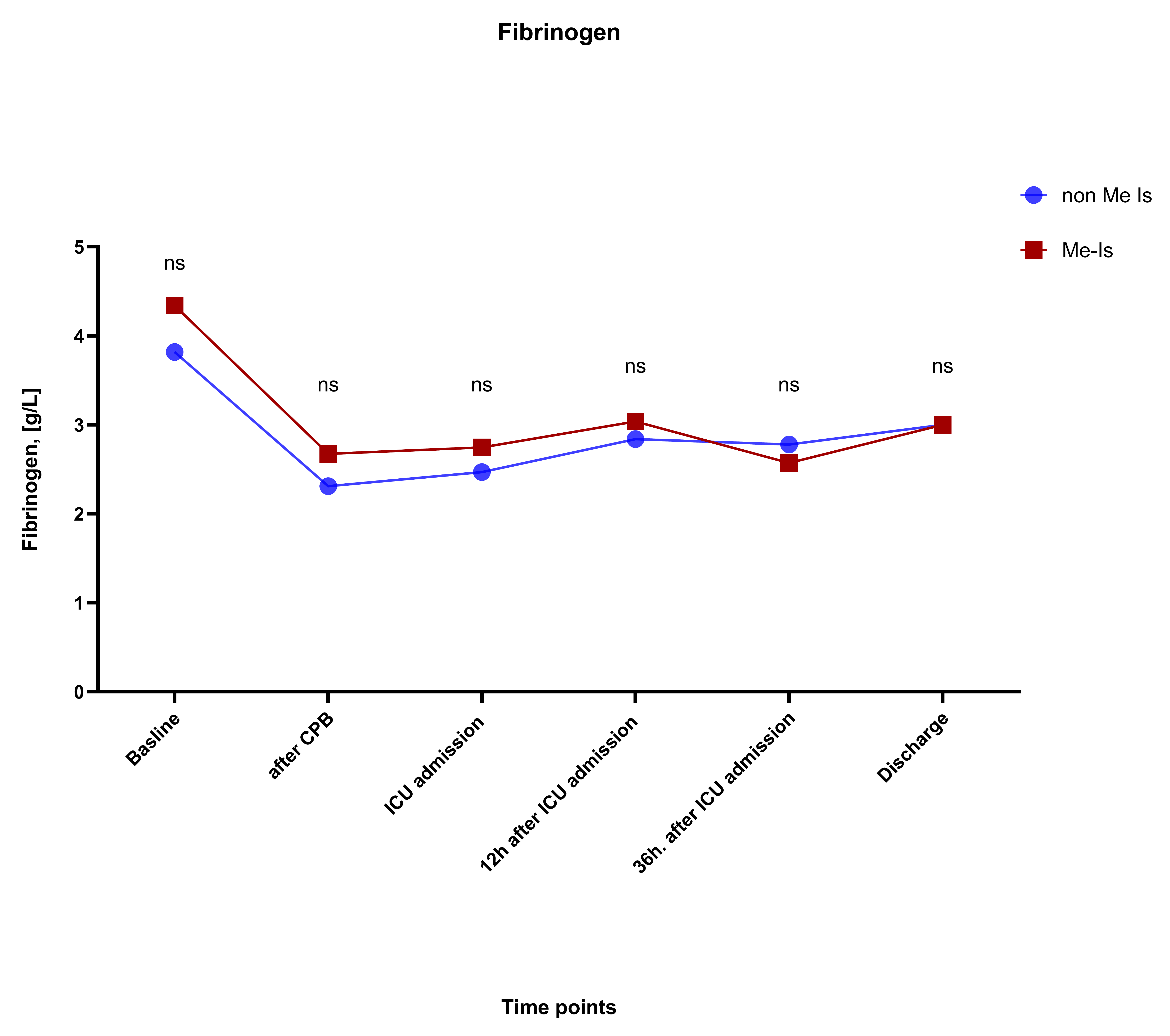

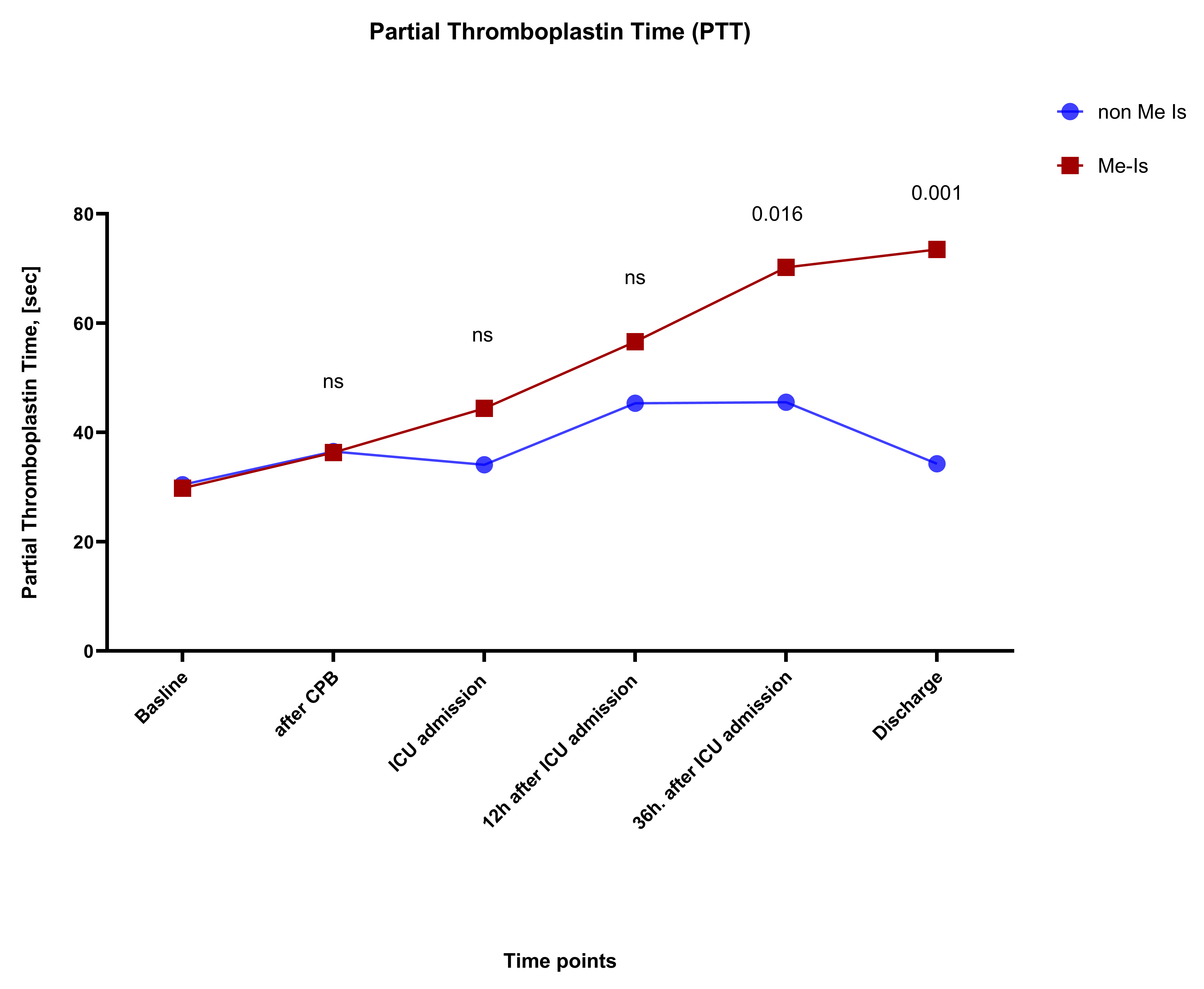

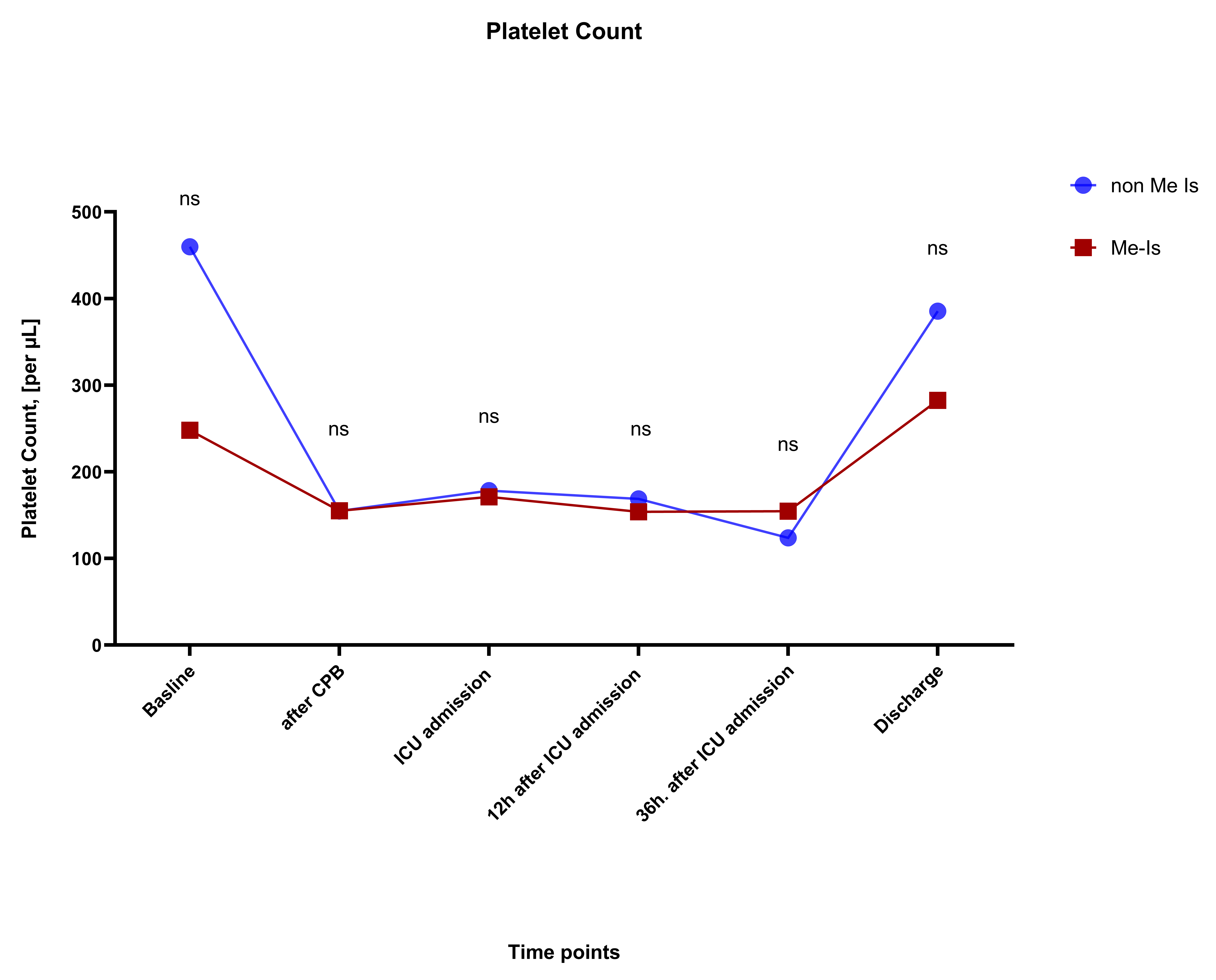


**Supplementary Figure 2.** Time course of fibrinogen, partial thromboplastin time (PTT), and platelet counts. Data are presented as mean ± SD. Statistical significance is indicated by p-values (p < 0.05).
